# Porphyrin-Based Metal-Organic Frameworks as Heterogeneous Catalysts in Oxidation Reactions

**DOI:** 10.3390/molecules21101348

**Published:** 2016-10-12

**Authors:** Carla F. Pereira, Mário M. Q. Simões, João P. C. Tomé, Filipe A. Almeida Paz

**Affiliations:** 1 Department of Chemistry & CICECO–Aveiro Institute of Materials, University of Aveiro, 3810-193 Aveiro, Portugal; carlafpereira@ua.pt; 2 Department of Chemistry & QOPNA, University of Aveiro, 3810-193 Aveiro, Portugal; jtome@ua.pt; 3 CQE, Instituto Superior Técnico, Universidade de Lisboa, Av. Rovisco Pais, 1049-001 Lisboa, Portugal

**Keywords:** metal-organic frameworks, porphyrins, oxidation, heterogeneous catalysis

## Abstract

Porphyrin-based Metal-Organic Frameworks (Por-MOFs) constitute a special branch of the wide MOF family that has proven its own value and high potential in different applications. In this mini-review the application of these materials as catalysts in oxidation reactions is highlighted.

## 1. Introduction

Heterogeneous catalysis constituted one of the earliest purposed applications for crystalline Metal-Organic Frameworks (MOFs) [1]. The MOF concept was introduced in the literature by Yaghi’s research group [2], being these materials, in the most elementary sense, obtained by connecting together metal ions with organic linkers, often resulting in fascinating structural architectures. We note that the term coordination polymer (CP) is more general, also including crystalline materials, assembled using the same basic principles, but which are non-porous [3].

Porphyrin-based MOFs (Por-MOFs) are composed by (metallo)porphyrin-based linkers interconnected by metal ions or metal cluster secondary building units (SBUs) [4]. These materials constitute a highly interesting branch of MOFs, which despite being in its infancy has, nevertheless, received much attention in the past decade. On the one hand, because porphyrins are structurally functional robust molecules with terminal pendant functional groups that can be easily tuned, while exhibiting a relatively rigid geometry and high thermal stability [5], resulting materials can thus be characterized by peculiar properties and architectures. On the other hand, the well-known role of metalloporphyrins in Nature in diverse biological functions as cofactors for many enzyme/protein families, including peroxidases, cytochromes, hemoglobins, and myoglobins [6,7], brings special importance for their use in a novel class of functional materials. For example, cytochrome P-450 (CP-450) features an iron porphyrin core and is well known in Nature for its performance in catalytic oxidations. In this sense, iron(III) porphyrins are typically used as models to mimic CP-450 in the catalytic oxidation of organic substrates [8,9,10,11]. The enzymatic pocket in CP-450 protects the porphyrin core preventing its decomposition and nonselective oxidation. Nevertheless, in a laboratory setting, because of the absence of the protective protein shell, metalloporphyrins can undergo catalytic deactivation *via* suicidal self-oxidation, lowering their catalytic activity and sustainability relatively to their counterparts in Nature [12,13,14]. It is, thus, easy to understand how the immobilization of (metallo)porphyrin moieties, by its utilization as linkers for the preparation of Por-MOFs materials, not only can retain but also enhance the functionalities of the individual building blocks. The main goal behind the immobilization of (metallo)porphyrin moieties in MOFs (or CPs) is thus very clear, consisting in the stabilization of these structures which ultimately facilitates the molecular recognition of substrates and the design of highly efficient catalysts.

In this sense, the preparation of MOFs using (metallo)porphyrins is currently highly attractive because it mimics diverse biological functionalities [15,16,17]. However, the preparation of catalytically active Por-MOFs still constitutes a great challenge because these materials often have large open pores, being thus more susceptible to structural collapse upon removal of the confined solvent molecules. It is also extremely difficult to incorporate free-base porphyrins as linkers because of their great tendency to scavenge and coordinate the metal ions present in the reaction media [18,19,20,21].

The first porphyrin-based CP was reported by Robson and co-workers back in 1991, a three-dimensional material composed of [tetrakis(*p*-pyridyl)porphyrinato] Pd(II) connected by Cd(II), which suffered an irreversible decomposition upon the removal of solvent because of its large cavities [22]. We note that this is an iconic paper in the realm of MOFs since, in a very simple fashion, the authors clearly describe many advantages and disadvantages of the MOF chemistry using a Por-MOF compound, which remained true to our days. The first catalytic study performed over a Por-MOF was reported 14 years later by Suslick et al. (details in the sections of alkanes and alkenes oxidation) for a three-dimensional net of [tetrakis(*p*-carboxyphenyl)porphyrinato] Mn(III) connected by trinuclear manganese clusters [23]. Besides the utilization of Por-MOFs as catalysts, namely as oxidative catalysts, the main subject explored and reviewed in the present document, these materials also revealed a high potential when used as photocatalysts and in the field of gas separation, storage and light harvesting [19,21].

## 2. Por-MOFs as Heterogeneous Catalysts in Oxidation Reactions

**Figure 1:**
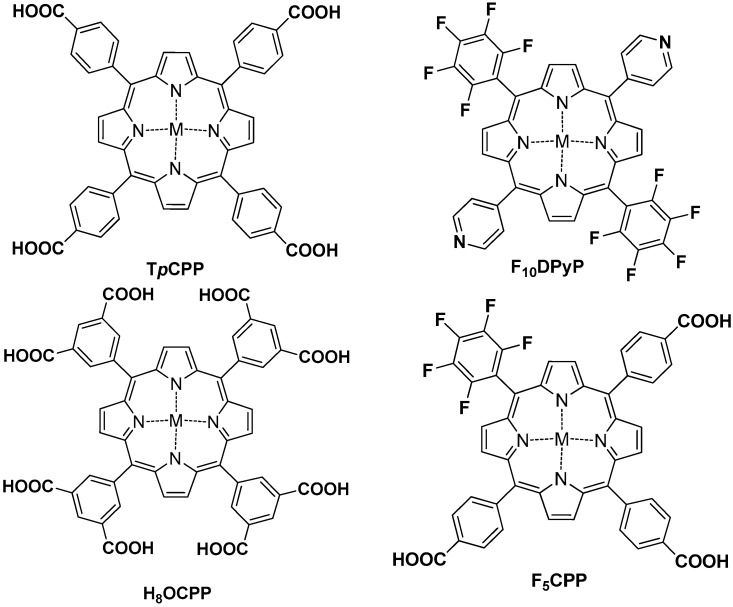

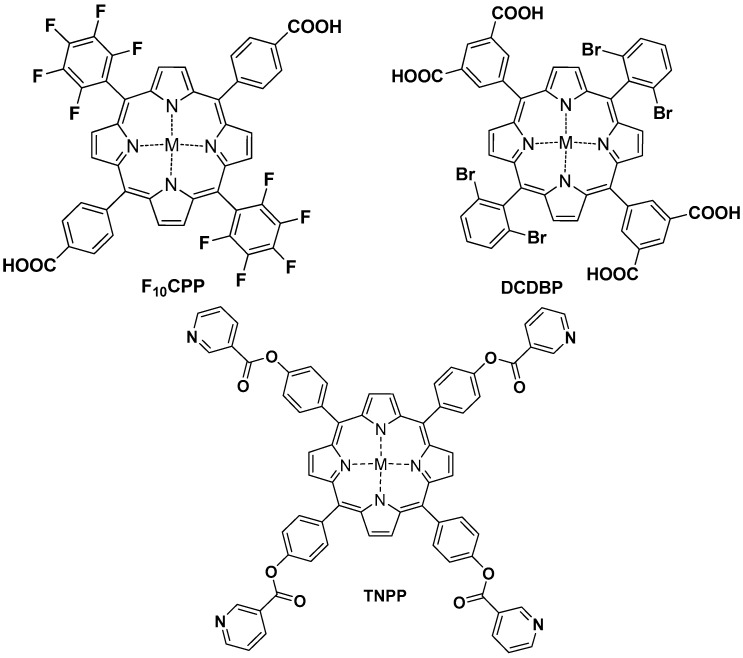
Porphyrins used in the preparation of Por-MOFs reported as catalysts in oxidation reactions.

Figure 1 depicts the structure of the porphyrins typically used as linkers in the preparation of the catalytically active Por-MOFs herein reviewed. Table 1 lists known catalytic Por-MOFs in oxidation reactions. A description of the substrates and informal mnemonic names (used in the description of the studies in the sections below) are also provided.

### 2.1. Oxidation of Alkanes

**Figure 2:**
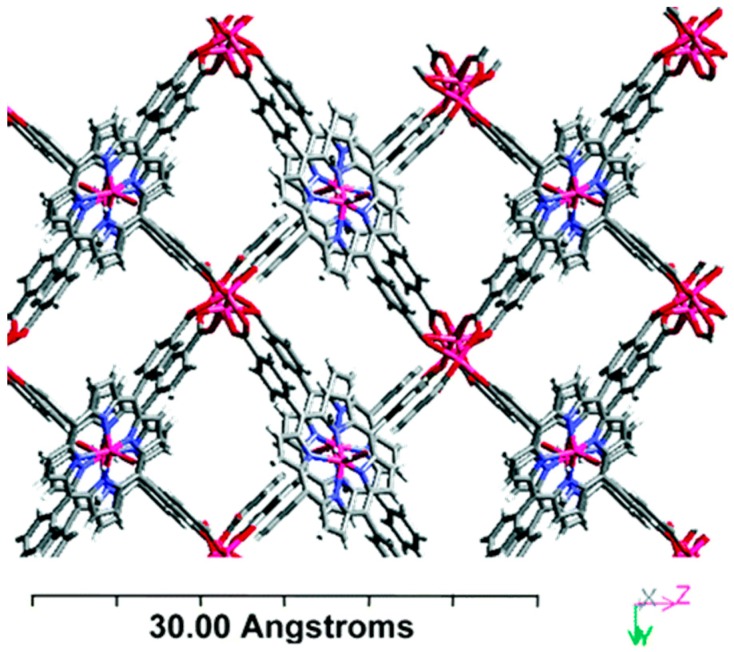
Schematic representation of the PIZA-3 Por-MOF viewed along the [1,0,0] direction of the unit cell. Solvent and coordinated *N*,*N*′-dimethylformamide molecules have been removed for clarity. Reprinted (adapted) with permission from [23]. Copyright (2005) American Chemical Society.

The first report of a catalytically active Por-MOF dates back to 2005 and concerns the PIZA-3 material (Porphyrinic Illinois Zeolite Analogue—PIZA, Figure 2). This compound consists of a three-dimensional net of [5,10,15,20-tetrakis(*p*-carboxyphenyl)porphyrinato] Mn(III) connected by trinuclear manganese clusters [23]. The compound is stable upon the removal of the solvent molecules, and catalytically active in the oxidation of linear and cyclic alkanes at ambient temperature using iodosylbenzene as oxidant. The small channels in these materials restricted the catalysis of larger substrates, being concluded by the researchers that the catalysis is dominated by the reactivity on the exterior surface of the crystallites. The oxidation of alkanes is selective for the alcohol in the case of cyclic substrates, with an alcohol/ketone ratio of 8.9 and 8.0, achieved for the cyclic substrates cyclohexane (43% yield of alcohol) and cycloheptane (47% yield of alcohol), respectively. On the other hand, the hydroxylation of the linear alkanes such as hexane (17% total alcohols: 2-ol; 3-ol; 4-ol) and heptane (23% total alcohols: 2-ol; 3-ol; 4-ol) catalyzed by PIZA-3 gives similar yields for alcohols at positions 2 and 3 [23].

Hupp, Farha and coworkers employed Zn paddlewheel nodes to build three-dimensional MOFs with open channels. Although paddlewheel clusters are known to be less stable in humid conditions, they succeeded in building a catalytically active MOF based on this node by employing a mixed-ligand strategy [24]. ZnMn-RPM (Robust Porphyrinic Material—RPM), catalytically competent for the oxidation of alkanes and alkenes, was prepared using a [5,10,15,20-tetrakis(*p*-carboxyphenyl)porphyrinato] Zn(II) as a spacer and the redox metalloligand [5,15-bis(pentafluoro-phenyl)-10,20-di(pyridyl)-porphyrinato] Mn(III) structured by paddlewheel dinuclear zinc(II) clusters [24].

**Figure 3:**
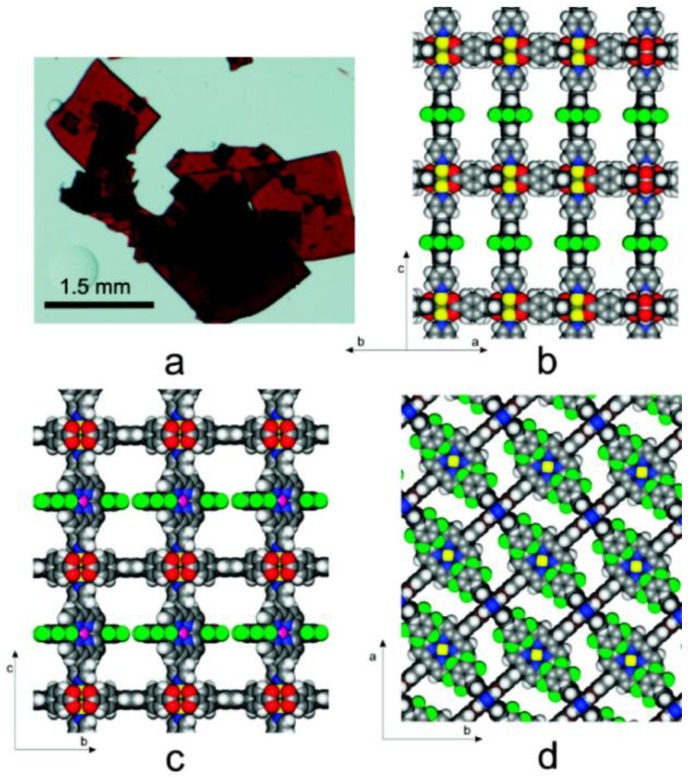
(**a**) Optical photograph of ZnMn-RPM crystals; (**b**–**d**) Schematic representations of the crystal structure features of ZnMn-RPM showing channels in the three crystallographic directions (Colour scheme: yellow = Zn, red = O, green = F, blue = N, gray = C, white = H, purple = Mn). Disordered solvent molecules have been omitted for clarity. Reprinted (adapted) with permission from [24]. Copyright (2011) American Chemical Society.

The choice of these porphyrins had a clear purpose: the dipyridyl porphyrin was chosen because of the ability of electron-withdrawing groups, such as fluorine, to greatly increase the activity of metalloporphyrins for oxidative catalysis. On the other hand, the choice of tetracarboxylated porphyrin was because of the design principles of MOFs, so to lead to the formation of the intended paddlewheel clusters and avoid the formation of interpenetrated networks. The structure of ZnMn-RPM is depicted in Figure 3.

**Scheme 1:**
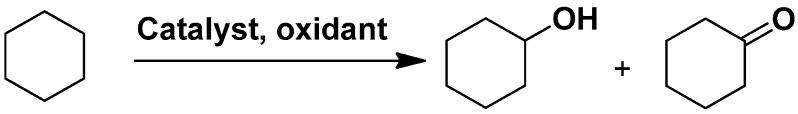
General scheme of cyclohexane oxidation.

In the oxidation of cyclohexane at 25 °C using 2-(*tert*-butylsulfonyl)iodosylbenzene as oxidant (Scheme 1), ZnMn-RPM led to a total yield of 20%, with 83% and 17% selectivity for cyclohexanol and cyclohexanone, respectively. The poor yield was, nevertheless, attributed to diffusion difficulties of the substrate into the solid catalyst [24].

Jiang and collaborators reported in 2014 [25] a new porphyrin-based MOF composed of manganese 5,10,15,20-tetrakis(*p*-carboxyphenyl)porphyrin as a bridging ligand bound to Fe(II) ions (Fe-MMOF). The material revealed to be an efficient catalyst in the selective oxidation of versatile natural substrates, acting as an effective peroxidase mimic. This compound is an efficient catalyst in the oxidation of different substrates, namely cyclohexane, different alkenes, cyclohexanol (*please note*: pertinent results for the oxidation of alkenes and alcohols will be presented and discussed in the sections below). Using this material as catalyst in the oxidation of cyclohexane led to a conversion up to 70%, and about 100% selectivity toward cyclohexanone.

**Figure 4:**
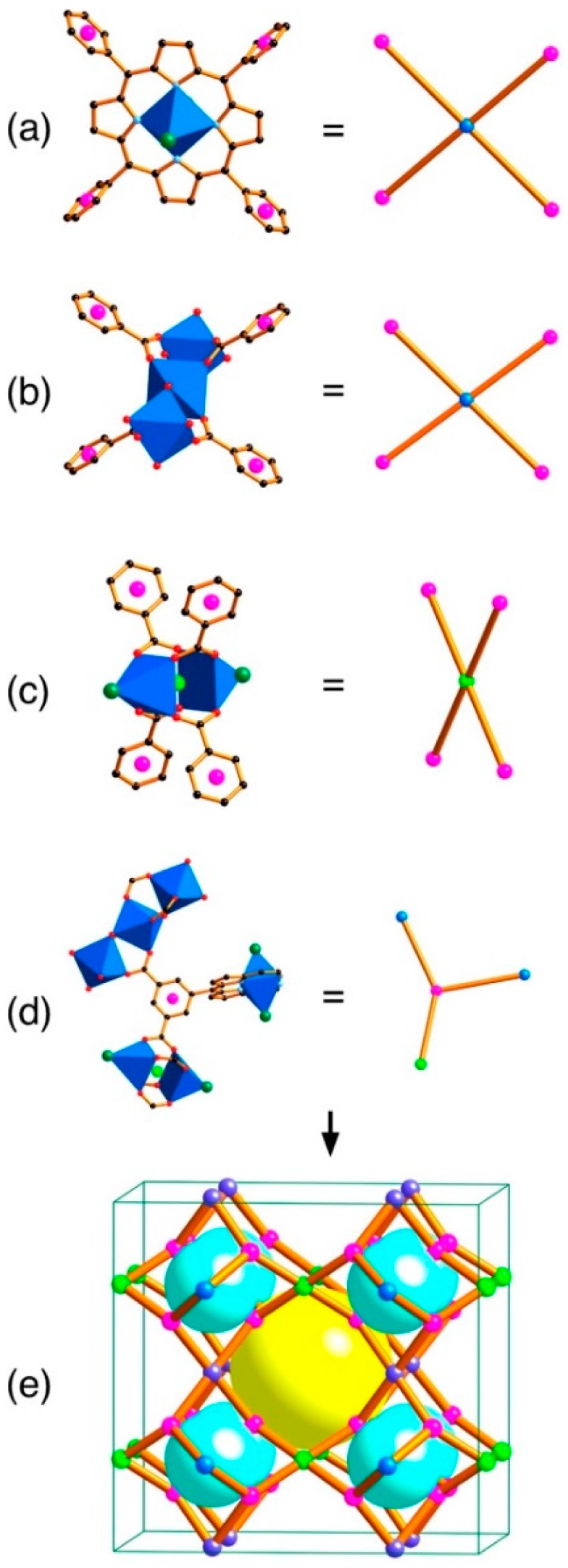
Mn-containing SBUs (**a**) Mn^III^N_4_Cl; (**b**) trinuclear [Mn_3_(COO)_4_(μ-H_2_O)_2_(H_2_O)_6_]; and (**c**) binuclear paddlewheel [Mn^II^_2_(COO)_4_Cl_2_] and (**d**) their linkage to the 3-coordinated node to form (**e**) the net from four nodes in the actual crystal structure of ZJU-18. Yellow and blue balls are centered in the large and small cavities in the structure. Reprinted (adapted) with permission from [26]. Copyright (2012) American Chemical Society.

To date there are, to the best of our knowledge, only a couple of reports focused on the oxidation of aromatic alkanes using Por-MOFs as catalysts [26,27]. The research groups of Chen and Wu described the utilization of 5,10,15,20-tetrakis(3,5-dicarboxylphenyl)porphyrin with different metals in the core as a powerful linker to the preparation of isotypical Por-MOFs with high porosity: ZJU-18, ZJU-19 and ZJU-20 (ZJU—Zheijang University). The incorporation of functional groups into the porphyrin synthons and its 3D configuration within the porous frameworks exhibited a synergistic effect for highly efficient oxidation of alkylbenzenes [26]. ZJU-18 is built up from a manganese porphyrin and manganese individual clusters (Figure 4), while ZJU-19 has nickel(II) porphyrin as building block and manganese SBUs; finally ZJU-20 consists of manganese porphyrins connected by cadmium clusters (see Table 1 for the individual empirical formulae). In the oxidation of ethylbenzene to acetophenone at 65 °C by *tert*-butyl hydroperoxide, the homometallic ZJU-18 was found to be efficient with quantitative conversion values. On the other hand, in the case of ZJU-19, a negligible catalytic activity was obtained with only 9% of acetophenone being formed. The significant difference in the catalytic transformation of ethylbenzene to acetophenone clearly showed that the manganese porphyrin sites in the material ZJU-18 are the efficient catalytic sites. On the other hand, the manganese sites on the SBU nodes might also be partially responsible and work collaboratively with the manganese porphyrin, improving the overall catalytic activity. Indeed, conversion values obtained for ZJU-20 are much higher (conversion of 69%) than those registered for ZJU-19.

The homogeneous catalysis with manganese (III) and nickel (II) porphyrins typically affords conversions to ketone of 16% or traces, respectively. In this way, these results showed that the building blocks of the manganese porphyrin clearly are the catalytically active species in the ZJU family, and that the Mn-SBU clusters contribute only slightly to oxidative reactions. In the specific case of ZJU-18, the material showed to be better than the catalyst in homogeneous phase because the MOF architecture avoids self-oxidation and dimerization of the metalloporphyrin.

The good performance of ZJU-18 prompted the researchers to study its activity in the oxidation of longer-chain alkylbenzenes. The compound with a larger alkyl chain presents the worst catalytic performance of this metalloporphyrinic MOF, suggesting that catalysis also depends on the accessibility to the channels of the material. The oxidation of diphenylmethane, for which ZJU-18 gives lower conversion when compared with the same reaction in homogeneous medium (18% and 26% conversion, respectively), confirms this assumption. This catalytic pattern is also true for the oxidation of 4-phenyl-ethylbenzene by ZJU-18 (conversion of 16% and 28% in heterogeneous and homogeneous medium, respectively), but not for smaller substrates. All these observations prompted the authors to conclude that catalysis occurs only at the external surface of the MOF for larger substrates, whilst it takes place inside the pores for smaller hydrocarbons. Therefore, ZJU-18 is size- and shape-selective [26].

**Scheme 2:**
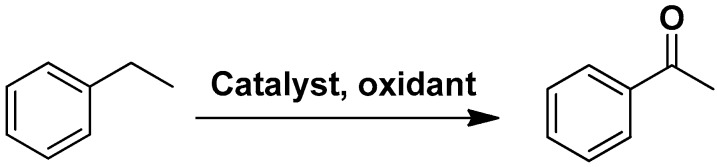
General scheme of ethylbenzene oxidation.

Li and co-workers described six Mn^III^(porphyrin)-based porous CPs in which 5,10,15-tris(*p*-carboxyphenyl)-20-(pentafluorophenyl)porphyrin manganese(III) [Mn^III^(F_5_CPP)] or 5,15-bis(*p*-carboxyphenyl)-10,20-bis(pentafluorophenyl)porphyrin manganese(III) [Mn^III^(F_10_CPP)] molecules were interlinked *via* coordination to the peripheral carboxylate groups of several transition metal centers: Mn^III^(F_5_CPP)–Mn^II^, Mn^III^(F_5_CPP)–Co^II^, Mn^III^(F_5_CPP)–Ni^II^, Mn^III^(F_10_CPP)–Mn^II^, Mn^III^(F_10_CPP)-Co^II^ and Mn^III^(F_10_CPP)-Ni^II^. The catalytic performance of these CPs was studied in the oxidation of ethylbenzene at 65 °C using *tert*-butyl hydroperoxide as oxidant (Scheme 2). All six materials revealed to be efficient as catalysts with acetophenone being obtained with yields between 71 and 82% and with selectivity over 99%. Authors justified the higher catalytic activity of the CPs over the homogeneous counterparts to the combined effect of the immobilization of the catalytic active units Mn(F_5_CPP)/Mn(F_10_CPP), thus preventing their self-dimerization and oxidative degradation, and to the fact that both of the Mn(F_5_CPP)/Mn(F_10_CPP) units and metal ions nodes were involved in the catalytic reaction, probably *via* a synergistic effect. Authors also reported the utilization of the CPs as catalysts for three consecutive times with only a slight decrease of the catalytic activities and without any detectable leaching of the catalysts [27].

### 2.2. Oxidation of Alkenes

**Scheme 3:**
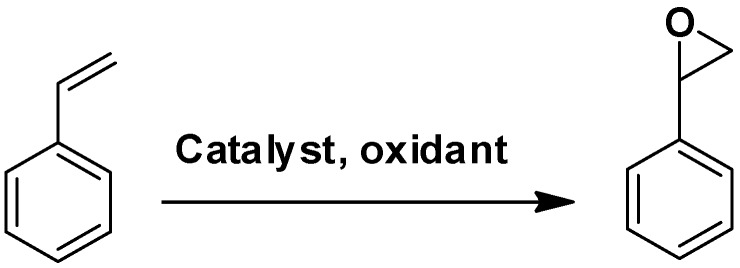
General scheme of styrene epoxidation.

The catalytically active Por-MOF (PIZA-3) mentioned in the previous subsections also proved to be active in the epoxidation of alkenes using iodosylbenzene as oxidant. This MOF selectively oxidizes cyclopentene (23% epoxide), cyclohexene (23% epoxide), cyclooctene (74% epoxide), and limonene (20% 1,2-epoxide), with no allylic products being detected after the performed reactions [23]. ZnMn-RPM also proved to be active in the epoxidation of styrene at 25 °C using 2-(*tert*-butylsulfonyl)iodosylbenzene as oxidant (Scheme 3) [24]. This Por-MOF is more stable when compared with the homogeneous counterpart [5,10,15,20-tetrakis-(pentafluorophenyl)-porphyrinato] Mn(III) chloride, a second-generation catalyst [37]. In fact, while this second-generation catalyst affords a turnover number (TON) of 780 in homogeneous catalysis, because of total catalyst deactivation, ZnMn-RPM affords a TON of 2150 after complete oxidant consumption. The catalysis is heterogeneous because after removal of the MOF itself the catalytic reaction stops. Moreover, no manganese porphyrin was leached during the reaction [24].

**Figure 5:**
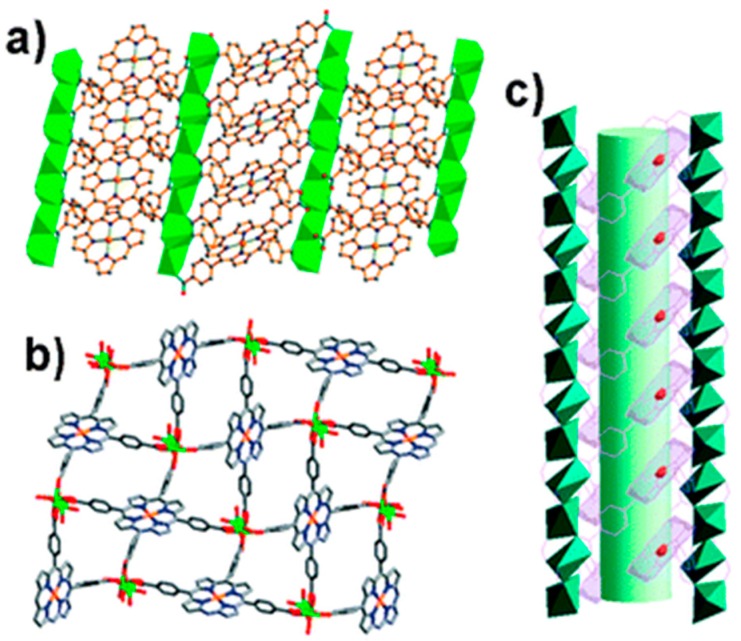
(**a**) Crystal packing of [Cd_1.25_(Pd−H_1.5_T*p*CPP)-(H_2_O)]·2DMF viewed along the [1,1,0] direction, depicting the arrangement of the palladium-porphyrins; (**b**) Schematic representation of the crystal structure of the material viewed down the [1,0,0] crystallographic direction, emphasizing the 1D opening channels and the accessible Pd^II^ sites; (**c**) Side view of the 1D channel present in this porous framework. Reprinted (“Adapted” or “in part”) from [28] with permission of the PCCP Owner Societies.

**Scheme 4:**
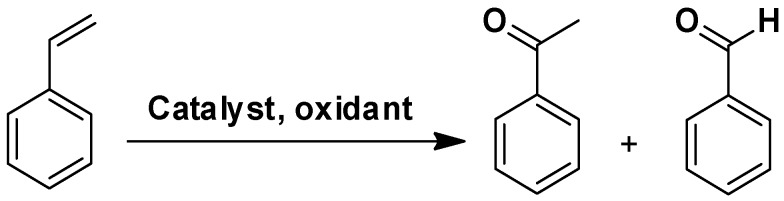
General scheme of styrene oxidation.

Also in 2011 Wu and coworkers reported the porous coordination network [Cd_1.25_(Pd−H_1.5_T*p*CPP)(H_2_O)]·2DMF, which comprises [5,10,15,20-tetrakis(*p*-carboxyphenyl)- porphyrinato] Pd(II) and Cd(II) connecting nodes (Figure 5). This material was studied as catalyst in styrene oxidation [28]. Results suggested that the square-coordinated Pd(II) ions in T*p*CPP are highly active in oxidation reactions by forming high valent Pd intermediates. In this sense, authors reported a complete oxidation of styrene into a mixture of 91% acetophenone and 9% benzaldehyde at 55 °C using H_2_O_2_ as oxidant (Scheme 4).

It was further demonstrated that the catalytic reaction occurs inside the pores of the material. Remarkably this MOF exhibits better results than those obtained with the metalloporphyrin in homogeneous medium (90% conversion and 78% selectivity for acetophenone) [28].

In the evaluation of the influence of different acids in the oxidation of styrene (Scheme 4), it was observed that acetic acid did not boost the catalytic transformation, while hydrochloric acid prompted the transformation less efficient. Despite the acetophenone selectivity values with nitric acid (86% for acetophenone and 14% for benzaldehyde) and sulfuric acid (95% for acetophenone and 5% for benzaldehyde) are comparable to that of perchloric acid (91% for acetophenone and 9% for benzaldehyde), the styrene substrate cannot be oxidized completely. This Por-MOF system proved to be heterogeneous with the catalyst being recovered and reused in subsequent cycles [28].

**Figure 6:**
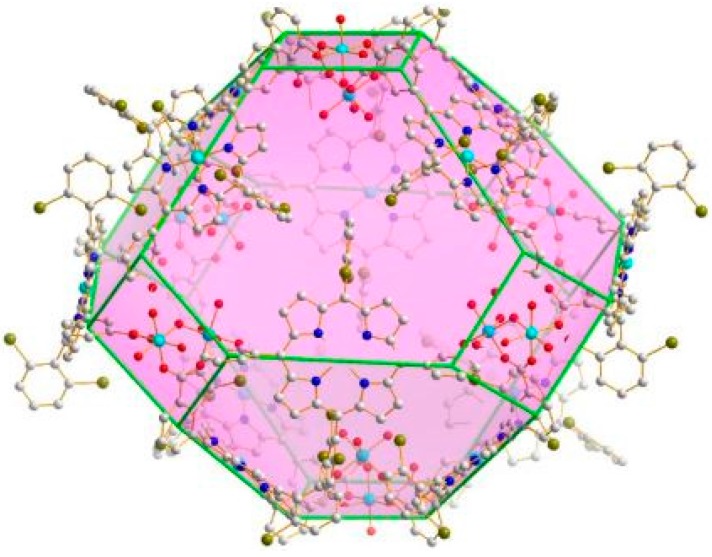
Schematic representation of the truncated octahedron polyhedral cages present in MMPF-3. Adapted from http://sqma.myweb.usf.edu/pages/publications.html#anchor2012.

**Scheme 5:**
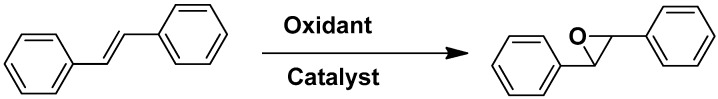
General scheme of of *trans*-stilbene epoxidation.

A family of metal-metalloporphyrin frameworks (MMPF) has been explored since 2011 by Ma´s research group [29,30,35,38], herein coined MMPF-2, -3, -5 and -6 (see Table 1 for additional details). These MOFs consist of carboxyphenyl-substituted metalloporphyrins linked through SBUs with different metals (Cu, Co, Zn, Cd, Zr, Fe) and do not contain any additional linkers or spacers. Noteworthy, different porphyrins were used for their construction: while for MMPF-2 and MMPF-5 [5,10,15,20-tetrakis(3,5-dicarboxyphenyl)porphyrinato] Co(III) was employed [30], the preparation of MMPF-3 and MMPF-6 involved, respectively, [5,15-bis(2,6-dibromophenyl)-10,20-bis(3,5-dicarboxyphenyl)porphyrinato] Co(III) [29] and [5,10,15,20-tetrakis(*p*-carboxyphenyl)-porphyrinato] Fe(III) [35]. Compound MMPF-3 exhibits different types of polyhedral cages – cubohemioctahedron, truncated tetrahedron and truncated octahedron, being the last one represented in Figure 6. When this material was used as catalyst in the epoxidation of *trans*-stilbene at 60 °C using *tert*-butyl hydroperoxide as oxidant (Scheme 5), a conversion and epoxide yield of ca. 96% and 87% were afforded, respectively.

Results were compared with the following control reactions: (i) homogeneous catalysis using only the cobalt porphyrin used as the MOF building block of MMPF-3, for which a conversion of 60% and a selectivity of 67% were obtained; (ii) reaction using another MOF with the same topology as MMPF-3, namely fcu-MOF-1, which contains the tetracarboxylic acid porphyrin as building block, for which a conversion of ca. 47% and a yield of ca. 77% were obtained; and (iii) a blank reaction with no catalyst, for which a low conversion of ca. 9% was registered. These results permit to conclude that the cobalt porphyrins account for the major catalytic activity of MMPF-3, but there is also some contribution from cobalt paddlewheel clusters because the topological analogous fcu-MOF-1 is also active in the same reaction [29].

**Figure 7:**
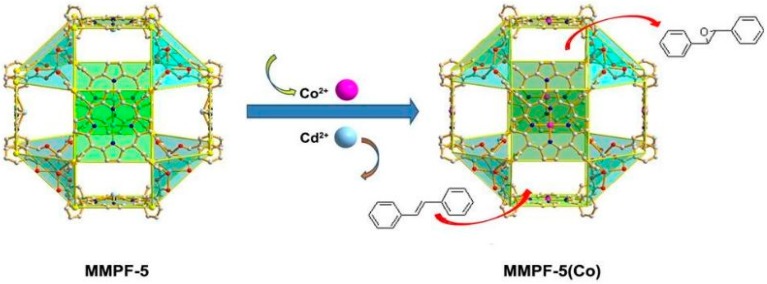
Schematic representation of MMPF-5(Co) preparation by post-synthetic modification, catalytically competent in *trans*-stilbene epoxidation. Adapted from http://sqma.myweb.usf.edu/pages/publications.html#anchor2013.

The other member of this family that also showed good activity as catalyst in the epoxidation of *trans*-stilbene was MMPF-5(Co). This material was prepared using post-synthetic modification by linker exchange from MMPF-5, which is a cadmium(II)-based MOF with [5,10,15,20-tetrakis(3,5-dicarboxyphenyl)porphyrinato] Cd(II) connected by cadmium(II) clusters (Figure 7). In this process, the cadmium porphyrins are exchanged for cobalt ones with the resulting MMPF-5(Co) framework retaining the crystal features of the parent MMPF-5 one.

MMPF-5(Co) catalyzes the oxidation of *trans*-stilbene into the epoxide with a conversion of ca. 87%, with the epoxide being obtained in ca. 82% yield. It is important to emphasize that the cadmium building units did not show any catalytic activity: control tests performed with MMPF-5 and without any catalyst typically led to very low conversions (both values of ca. 9%) [30].

Iron porphyrins are expected to be attractive building blocks for the design of catalytically active MOFs. Indeed, these could provide a solid basis to induce biomimetic catalysis. An identical rationale could be applied to manganese porphyrins, which are the basis of the heme cofactor analogues of CP-P450 enzymes that can efficiently oxidize a variety of organic molecules. Wu and coworkers reported four isotypical Por-MOFs based on tetrakis(*p*-carboxyphenyl)porphyrin which were used as catalysts in the epoxidation of olefins. The crystal structure of [Zn_2_(HCOO)(Fe^III^(H_2_O)-T*p*CPP)]·3DMF·H_2_O is composed of a layered network of Fe^III^-T*p*CPP connected with binuclear Zn_2_(COO)_4_ paddle-wheel SBUs, which are further linked by formate pillars to interconnect Zn_2_(COO)_4_ SBUs.

When Cd^2+^ ions are used as connecting nodes, two Fe^III^−HT*p*CPP ligands are coupled by one *μ*_2_-oxo group to form a dimer leading to the 3D microporous framework [Cd_3_(H_2_O)_6_(μ_2_-O)(Fe^III^-HT*p*CPP)_2_]·5DMF. These materials revealed to be inefficient catalysts in the epoxidation of styrene at ambient temperature using iodosylbenzene as oxidant, being obtained 17.9% and 8.4% yields of epoxide for [Zn_2_(HCOO)(Fe^III^(H_2_O)-T*p*CPP)]·3DMF·H_2_O and [Cd_3_(H_2_O)_6_(μ_2_-O)(Fe^III^-HT*p*CPP)_2_]·5DMF, respectively. This lack of efficiency was attributed to the self-oxidation of the individual pyrrolic rings. This fact was evident from bleaching studies [31]. When manganese porphyrins were used as synthons for the preparation of the materials, researchers observed that the axial coordination sites of the octahedrally coordinated Mn^III^ ions could be easily replaced by additional ligands, a feature that could obstruct the catalytic ability. The active Mn^III^ sites in the isotypical Por-MOFs [(CH_3_)_2_NH_2_][Zn_2_(HCOO)_2_(Mn^III^-T*p*CPP)]·5DMF·2H_2_O and [(CH_3_)_2_NH_2_][Cd_2_(HCOO)_2_-(Mn^III^-T*p*CPP)]·5DMF·3H_2_O were blocked by formate ligands, leading to inaccessibility of the active sites to the substrate. In this way, catalysis occurred on the external surface of the Por-MOF crystals, being afforded 100% product yield during styrene epoxidation. Other substrates were also tested as described in Table 1, being observed that these materials are efficient in the epoxidation of olefins at ambient temperature using iodosylbenzene as oxidant [31].

**Figure 8:**
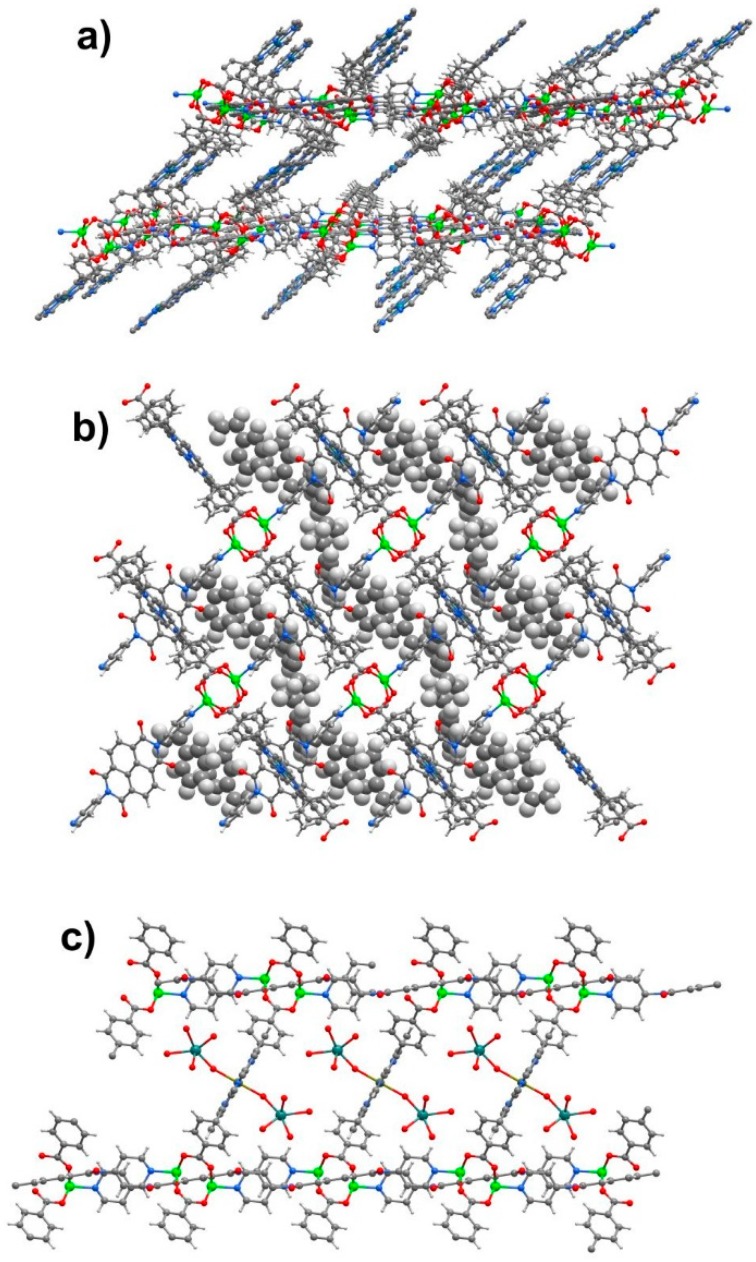
Crystal packing features of compounds (**a**) CZJ-1 viewed along [0,1,0] direction; (**b**) CZJ-1⊃2 styrene viewed along [0,0,1] direction; and (**c**) CZJ-1-Ti viewed along [0,1,0] direction. (Colour scheme: bright green = Zn, dark yellow = Mn, dark green = Ti, gray = H, dark gray = C, light blue = N, red = O).

The research groups of Chen and Wu described a porous Por-MOF coined as CZJ-1 (CZJ-Chemistry Department of Zhejiang University) based on a carboxylate Mn(III) porphyrin, *N*,*N*′-di(4-pyridyl)-1,4,5,8-naphthalenetetracarboxydiimide, and Zn(II) (Figure 8) which was highly efficient as catalyst in styrene epoxidation at ambient temperature using iodosylbenzene as oxidant (> 99% conversion and 98% selectivity). Authors proved that the reaction takes place inside the pores based on single-crystal X-ray diffraction studies of CZJ-1-Mn⊃2styrene (Figure 8b), and attributed the high catalytic efficiency of CZJ-1-Mn to a combination of factors: neighboring Mn^III^-porphyrins, flexibility of the structure and specific electronic environments around the catalytic Mn^III^ sites [32].

The efficient Fe-MMOF material reported by Jiang and coworkers in 2014 [25] also exhibited a high catalytic activity in the epoxidation of different alkenes at ambient temperature using hydrogen peroxide as oxidant and NaHCO_3_ as co-catalyst. Under these conditions, cyclooctene was almost fully oxidized into the epoxide with 99% selectivity, registering a very high conversion (>99%) and selectivity (>99%) until the third cycle. For the epoxidation of cyclohexene a conversion of 87% and a selectivity of >99% were afforded instead. The authors further described a conversion of 99% for the epoxidation of styrene.

The low electron density of styrene and methyl acrylate usually reduces their nucleophilicity toward electrophilic oxygen of porphyrin-Mn^V^ = O. Based on the comparison of the catalytic performance of Fe-MMOF with their primary building blocks (MnCl_2_, FeCl_2_ and Mn-T*p*CPP), as well as with that of the physical mixtures obtained under identical reaction conditions, authors postulated that the active catalytic sites should be the Mn-porphyrin moieties lining the pores of the Fe-MMOF network. The μ-oxo dimerization of Mn-porphyrin was prevented by its immobilization in the Fe-MMOF. Unlike the homogeneous porphyrin catalysts, Fe-MMOF could be easily recovered and reused [25].

**Figure 9:**
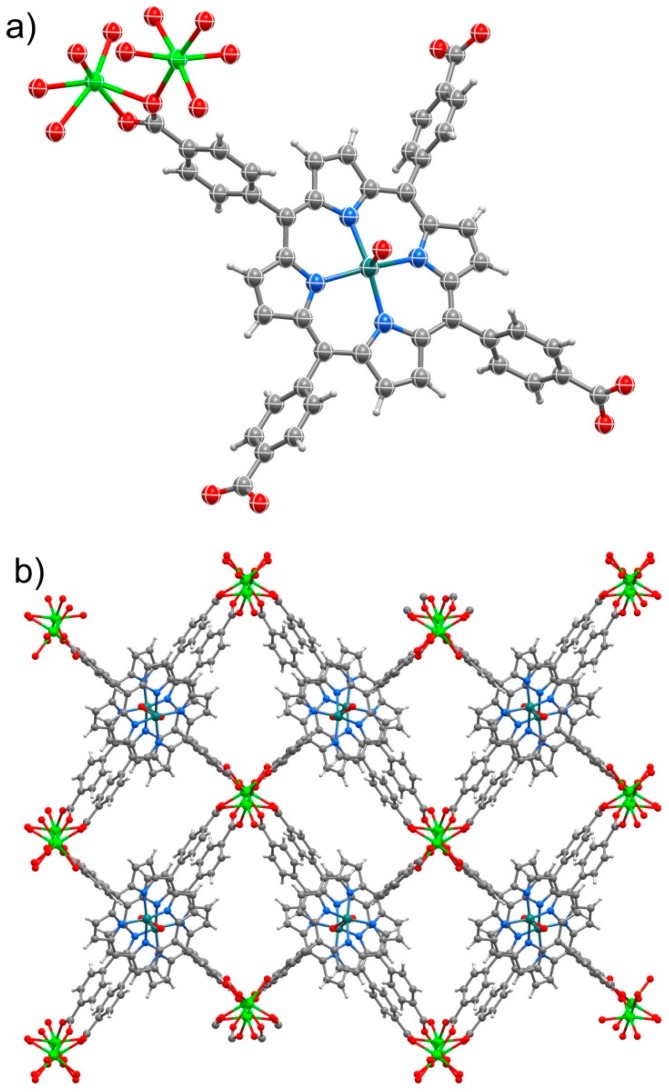
Schematic representation of the (**a**) asymmetric unit and (**b**) the crystal packing of MMPF viewed along [1,0,0] direction. (Colour scheme: bright green = Fe, dark yellow = Mn, gray = H, dark gray = C, light blue = N, red = O).

Jiang and collaborators reported a novel Por-MOF containing a Mn(III) tetrakis(*p*-carboxyphenyl)porphyrin as bridging ligand and Fe(III) as the metal source. This material, coined as MMPF (MMPF denotes iron-based Metalloporphyrinic Metal-Organic Framework) was successfully obtained as a 3D MOF (Figure 9). Authors described that MMPF is structurally robust in boiling water and when in contact with basic aqueous solutions with pH ranging from ca. 7 to 12. In acidic conditions MMPF decomposes into the metalloporphyrinic monomers. The solvent-induced breathing effect of MMPF was also demonstrated with the immersion of the material in different solvents [33]. The catalytic activity of MMPF was tested for the selective epoxidation of a variety of alkenes, as summarized in Table 1, using iodosylbenzene as oxidant at ambient temperature. It was observed that for cyclohexene, a conversion of 100% was obtained, while for cyclooctene a conversion of 93% was registered instead, both with a selectivity >99%. Based on these results, the authors postulated that MMPF contains channels with accessible catalytic sites for the substrates, which greatly facilitates their diffusion. In addition, the low electron density of conjugated alkenes usually reduces their nucleophilicity towards the electrophilic oxygen of porphyrin-Mn^V^ = O. The conversion of styrene and *trans*-stilbene is thus low, with only 50% selectivity for the epoxide being registered during the oxidation. Concerning the kinetic profiles, cyclohexene prompted a faster reaction, with the registered conversion being accordingly to the following order: cyclohexene > cyclooctene > hex-1-ene> styrene > *trans*-stilbene > oct-1-ene > dodec-1-ene [33].

In a similar fashion to that previously described for Fe-MMOF, based on a direct comparison of the catalytic activity of MMPF with its primary building blocks, as well as with the obtained physical mixtures prepared using identical reaction conditions, authors also concluded that for this system the Mn(II) porphyrins are the active catalytic sites. This catalytic process is heterogeneous in nature with MMPF being used in successive catalytic runs. Data show a constant high catalytic activity with a small decrease in the initial reaction rate for the oxidation of cyclooctene.

### 2.3. Oxidation of Phenols

The Fe-MMOF material reported by Jiang and coworkers in 2014 [25] is also active in the oxidation of phenols (Table 1). Among the tested substrates, benzyl alcohol reacted easier (99% conversion) in comparison with the other primary or secondary alcohols containing electron donating groups. Authors attributed the great versatility of this catalyst to the high-quality of the crystals, the stability of the framework and the pore structure having suitable pore sizes and catalytic metal sites with desirable electronic environments [25].

Polyphenolic compounds are commonly selected as substrates to perform the evaluation of the peroxidase activity of porphyrin catalysts. Three reports on Por-MOFs as catalysts in the oxidation of this type of compounds can be found in the literature, one using 3,5-di-*tert*-butylcathecol as substrate [34] while the other two employ 1,2,3-trihydroxybenzene instead [35,36].

**Scheme 6:**
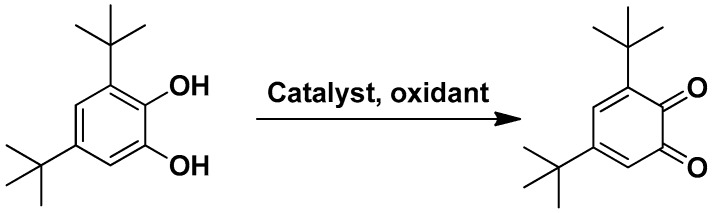
General scheme of 3,5-di-*tert*-butylcathecol oxidation.

**Figure 10:**
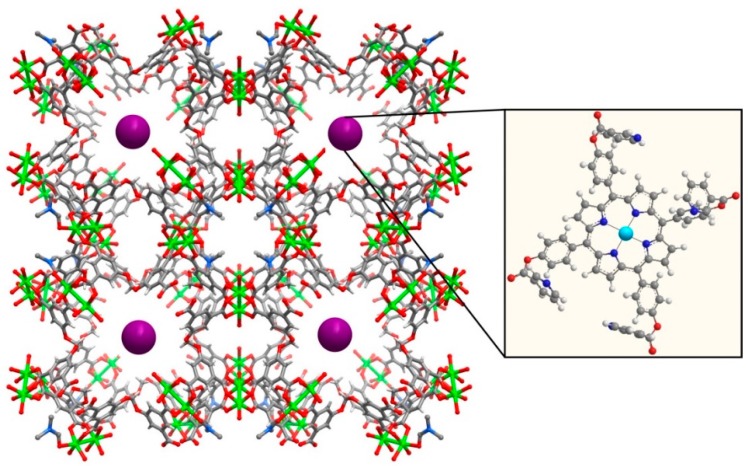
Schematic representation of porphyrin@MOFs type-catalysts. (Colour scheme: bright green = Cu, gray = H, dark gray = C, light blue = N, red = O, sky blue = metal in the porphyrin core, purple = porphyrin representation).

In the oxidation of 3,5-di-*tert*-butylcathecol by hydrogen peroxide at ambient temperature (Scheme 6), two new microporous materials were used as catalysts: CuTNPP@MOF and MnTNPP@MOF. These materials were prepared by the reaction of the flexible linker 1,1-bis-[3,5-bis(carboxy)phenoxy]methane and CuCl_2_ in the presence of 5,10,15,20-tetrakis[*p*-(nicotinoyloxy)-phenyl]porphyrin (TNPP) to afford CuTNPP@MOF or, in the presence of the manganese complex of TNPP(MnTNPP), to afford MnTNPP@MOF. In Figure 10 is represented the structure of these porphyrin@MOFs type-catalysts.

Authors observed that CuTNPP@MOF exhibited the highest catalytic activity with a high conversion of 3,5-di-*tert*-butylcathecol to the corresponding *o*-quinone (around 60%). In this case, the co-catalytic contribution of the nicotinoyl groups in CuTNPP was studied by performing the oxidation of 3,5-di-*tert*-butylcathecol in the presence of *meso*-tetrakis[4-methoxyphenyl]porphyrin. Because the transformation of the substrate was similar when using these compounds as catalysts, the catalytic activity of CuTNPP was assigned to the copper ion and not to any nicotinoyl functional groups in the periphery of the porphyrinic ring. In the recycling experiments, the catalytic activity of CuTNPP@MOF was maintained during four cycles. The oxidation of 3,5-di-*tert*-butylcathecol in the presence of MnTNPP@MOF led to a conversion not higher than 50%. In this case, conversion was not enhanced by the incorporation of MnTNPP, which in fact typically leads to a low conversion, very much similar to that obtained for the blank experiment [34].

**Figure 11:**
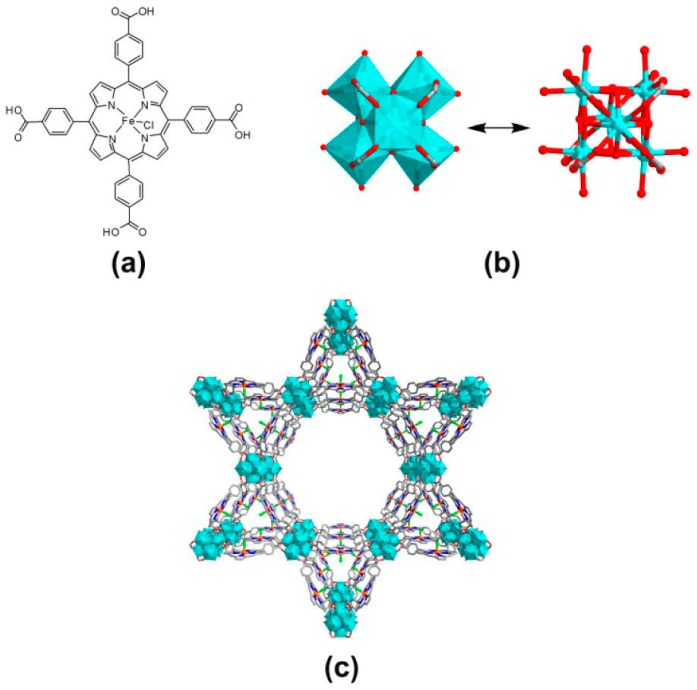
Schematic representations of the (**a**) iron(III) T*p*CPP chloride ligand, the (**b**) Zr_6_O_8_(CO_2_)_8_(H_2_O)_8_ SBUs and the (**c**) hexagonal and triangular one-dimensional channels of the crystal structure of MMPF-6. Color scheme: C, gray; O, red; Cl, green; Zr, turquoise. Reprinted (adapted) with permission from [35]. Copyright (2012) American Chemical Society.

**Scheme 7:**
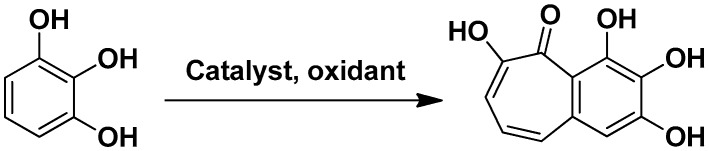
General scheme of 1,2,3-trihydroxybenzene oxidation.

MMPF-6 previously mentioned in the Section 2.2, constructed *via* the self-assembly of the [tetrakis(*p*-carboxyphenyl)porphyrinato] iron(III) chloride ligand with in situ generated Zr_6_O_8_(COO)_8_(H_2_O)_6_ SBUs (Figure 11), was further examined as catalyst in the peroxidase activity experiments commonly used to assess the biomimetic activity of synthetic biocatalysts. Both reactions involve the oxidation of a substrate: for the first, the oxidation of 1,2,3-trihydroxybenzene (Scheme 7) specifically examines the catalyst’s capability to transfer oxygen; in the second reaction, the oxidation of 2,2′-azino-bis(3-ethylbenzothiazoline)-6-sulfonate examines the efficiency of the catalyst in electronic transference. Authors reported an interesting peroxidase-like activity of MMPF-6, very much comparable to that of the heme protein myoglobin with respect to the initial reaction rates in buffer solution [35].

**Figure 12:**
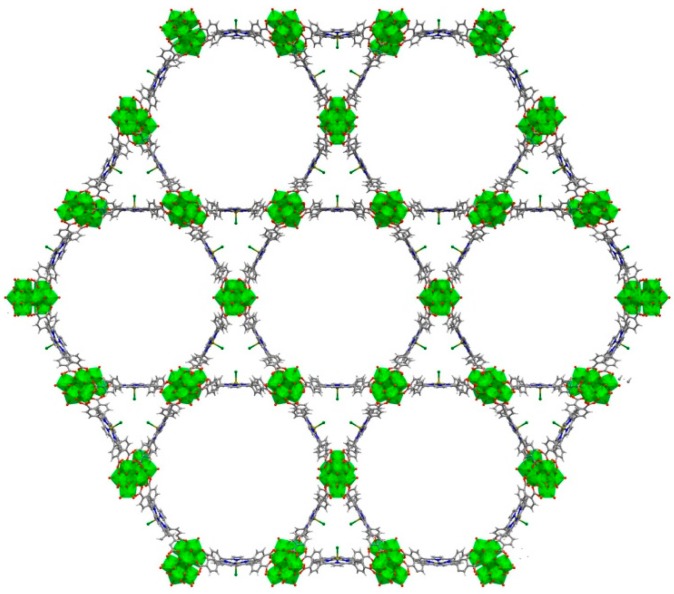
Schematic representation of the 3D network in Kagome-like topology of PCN-222(Fe) viewed along [0,0,1] direction. (Colour scheme: bright green = Zr, dark yellow = Fe, green = Cl, gray = H, dark gray = C, light blue = N, red = O,).

Simultaneously, Zhou and co-workers reported a stable Por-MOF, coined as PCN-222(Fe), prepared from ZrCl_4_ and Fe-T*p*CPP in the presence of benzoic acid (Figure 12). The zirconium(IV) cation is responsible for the high stability of the network. Indeed, due to its high charge density, the this cation polarizes the oxygen atoms of the carboxylate groups to form strong Zr—O bonds with a significant covalent character. The resulting PCN-222(Fe), also having iron centers, exhibited excellent peroxidase-like catalytic activity, whereas other MOFs did not show significant properties under identical conditions, which proves the stability of PCN-222 as an efficient, accessible biomimetic catalyst. The authors further reported an enzymatic kinetic study for PCN-222(Fe), being observed that the *ĸ*_cat_ value of the PCN-222(Fe) catalyst is 16.1 min^−1^, which is approximately seven times higher than the value for free hemin (2.4 min^−1^) (*please note*: *ĸ*_cat_ gives the maximum number of substrate molecules catalyzed per molecule of catalyst per unit of time). On the other hand, the *K*_m_ value (≈0.33 mm) is lower than that of the natural enzyme HRP (horseradish peroxidase, 0.81 mm), which indicates a better affinity of the substrate to PCN-222(Fe) (*please note*: *K*_m_ is the Michaelis constant and indicates the affinity of the catalyst molecules for the substrate). Other substrates such as 3,3′,5,5′-tetramethylbenzidine and *o*-phenylenediamine were also tested for peroxidase-like oxidations to demonstrate the general applicability of PCN-222(Fe) as an enzyme mimic. PCN-222(Fe) showed superior catalytic activity over free hemin as its *K*_Cat_ value was nearly ten times higher than that of free hemin [36].

## 3. Outlook

The unique physicochemical properties and the large diversity of porphyrin derivatives make them an ideal class of linkers for the design and preparation of new crystalline frameworks. Nevertheless, despite the intensive individual research over the years on both porphyrins and MOFs, the combination of both research worlds to produce novel materials (coined in this review as Por-MOFs) is still in an early stage. The delay of 14 years between the first report of a Por-MOF and the study of the potential of this branch of materials as catalysts clearly demonstrates the difficult challenges found by researchers concerning their preparation. It is possible to conclude by the scrutiny of the construction units employed that the porphyrins bearing carboxylic/pyridyl moieties and the transition metals used in the first reports still dominate the niche of Por-MOFs. Because of the high potential of this novel class of compounds, clearly demonstrated in the present short literature review, combined with new high-yield synthetic methodologies for both MOFs and porphyrins, it is expected that a more profound research will be carried out in the near future in the use of porous heterogeneous Por-MOFs as catalysts. In the addition the use of modern and more advanced X-ray laboratory equipment able to provide a faster response in the characterization of these materials, has significantly contributed to the characterization of these “giant” and new materials.

The inclusion of porphyrins as linkers in Por-MOFs, as well as its incorporation into porous MOFs, presents many advantages in the catalytic performance of these materials due to several reasons: (i) isolation of the catalytic centers that usually suffer degradation in solution through undesirable reactions; (ii) Por-MOFs usually exhibit large pores that lead to the fast diffusion of molecules; (iii) good interaction between open metal sites and substrates and (iv) the catalytic activity of the porphyrinic linkers can be adjusted with the study of different metals coordinated to the porphyrinic core.

Throughout this review it was possible to infer that in the last years there is a clear concern to perform adequate control experiments instead of just report that a certain Por-MOF is catalytically competent. Even in this way, the lack of mechanistic studies in this branch of materials is very clear. In addition to this lacuna, and because the nature of the oxidant is an important factor in an oxidation reaction, an appropriate screening of the most effective oxidants in each case remains pertinent. The knowledge gathered to date in the research of porphyrins as oxidative catalysts, under homogeneous and heterogeneous conditions, has undoubtedly a very high value, but the screening for the more adequate conditions to use in oxidation reactions with Por-MOFs as catalysts needs to be performed.

We are firm believers that this is an exciting new research area which will see a fast growth in coming years. We envision the preparation of ultra-stable Por-MOFs isolated from porphyrins with increased stability in comparison with the porphyrins nowadays used as building blocks. We believe that the future of this niche will lie in their study mainly as artificial enzymes in order to overcome the main problems, mostly related to stability, found over many years in the research on porphyrins as biomimetic catalysts.

## Figures and Tables

**Table 1:** Por-MOFs reported as catalysts in oxidation reactions.

MOF *^a^*	Catalyzed Reaction (s)	Substrate (s)	Ref.
[(Mn(T*p*CPP)Mn_1.5_)(C_3_H_7_NO)]·5C_3_H_7_NO (**PIZA-3**)	Oxidation of linear and cyclic alkanes	cyclohexane, cycloheptane, hexane and heptane	[23]
	Epoxidation of olefins	cyclopentene, cyclohexene, cyclooctene and limonene	
ZnMn-RPM (Zn designates the metal in T*p*CPP while Mn designates the metal in F_10_DPyP)	Oxidation of cyclic alkanes	cyclohexane	[24]
	Epoxidation of olefins	styrene	
Fe-MMOF	Oxidation of alkanes	cyclohexane	[25]
	Epoxidation of olefins	cyclooctene,cyclohexene, styrene, hex-1-ene, oct-1-ene, dodec-1-ene, 1*H*-indene, vinyl acetate, methyl acrylate	
	Oxidation of alcohols	cyclohexanol, benzyl alcohol, octan-2-ol, pentan-1-ol	
[Mn_5_Cl_2_(MnCl-OCPP)(DMF)_4_(H_2_O)_4_]·2DMF·8CH_3_COOH·14H_2_O (**ZJU-18**)	Oxidation of aromatic alkanes	ethylbenzene, diphenylmethane, 4-phenyl-ethylbenzene	[26]
[Mn_5_Cl_2_(Ni-OCPP)(H_2_O)_8_]·7DMF·6CH_3_COOH·11H_2_O (**ZJU-19**)		ethylbenzene	
[Cd_5_Cl_2_(MnCl-OCPP)(H_2_O)_6_]·13DMF·2CH_3_COOH·9H_2_O (**ZJU-20**)			
Mn^III^(F_5_CPP)–Mn^II^	Oxidation of aromatic alkanes	ethylbenzene	[27]
Mn^III^(F_5_CPP)–Co^II^			
Mn^III^(F_5_CPP)–Ni^II^			
Mn^III^(F_10_CPP)–Mn^II^			
Mn^III^(F_10_CPP)-Co^II^			
Mn^III^(F_10_CPP)-Ni^II^			
[Cd_1.25_(Pd−H_1.5_T*p*CPP)-(H_2_O)]·2DMF	Oxidation of alkenes	styrene	[28]
[Co_2_(μ_2_-H_2_O)(H_2_O)_4_](Co-DCDBP) (**MMPF-3**)	Epoxidation of olefins	*trans*-stilbene	[29]
[Cd_8_(Cd-OCPP)_3_][(H_3_O)_8_] (**MMPF-5**)	Epoxidation of olefins	*trans*-stilbene	[30]
[(CH_3_)_2_NH_2_][Zn_2_(HCOO)_2_(Mn^III^-T*p*CPP)]·5DMF·2H_2_O	Epoxidation of olefins	styrene, cyclopentene, cyclohexene, cyclooctene, terminal linear alkenes (hex-1-ene, oct-1-ene, dodec-1-ene), stilbene, and some modified stilbenes and styrenes	[31]
[(CH_3_)_2_NH_2_][Cd_2_(HCOO)_2_(Mn^III^-T*p*CPP)]·5DMF·3H_2_O			
[Zn_2_(HCOO)(Fe^III^(H_2_O)-T*p*CPP)]·3DMF·H_2_O		styrene	
[Cd_3_(H_2_O)_6_(μ_2_-O)(Fe^III^-HT*p*CPP)_2_]·5DMF		styrene	
[Zn_2_(MnOH-T*p*CPP)(DPNI)]·0.5DMF·EtOH·5.5H_2_O (**CZJ-1**)	Epoxidation of olefins	styrene	[32]
MMPF	Epoxidation of olefins	cyclohexene, cyclooctene, hex-1-ene, oct-1-ene, dodec-1-ene, styrene, *trans*-stilbene	[33]
MnTNPP@MOF	Oxidation of alcohols	3,5-di-*tert*-butylcathecol	[34]
Zr_6_O_8_(CO_2_)_8_(H_2_O)_8_-[FeCl(T*p*CPP)] (**MMPF-6**)	Oxidation of alcohols	1,2,3-trihydroxybenzene	[35]
Zr_6_(OH)_8_-FeT*p*CPP (**PCN-222 (Fe)**)	Oxidation of alcohols	1,2,3-trihydroxybenzene, 3,3′,5,5′-tetramethylbenzidine, *o*-phenylenediamine	[36]

**^a^**T*p*CPP—5,10,15,20-tetrakis(*p*-carboxyphenyl)porphyrin;

F_10_DPyP—5,15-bis(pentafluorophenyl)-10,20-di(pyridyl)porphyrin;

H_8_OCPP—5,10,15,20-tetrakis(3,5-dicarboxyphenyl)porphyrin;

F_5_CPP—5,10,15-tris(*p*-carboxyphenyl)-20-(pentafluorophenyl)porphyrin;

F_10_CPP—5,15-bis(*p*-carboxyphenyl)-10,20-bis(pentafluorophenyl)porphyrin;

DCDBP—5,15-bis(3,5-dicarboxyphenyl)-10,20-bis(2,6-dibromophenyl)-porphyrin;

DPNI—*N,N*′-di(4-pyridyl)-1,4,5,8-naphthalenetetracarboxydiimide;

TNPP—5,10,15,20-tetrakis[*p*-(nicotinoyloxy)phenyl]porphyrin.

## Abbreviations

The following abbreviations are used in this manuscript:

CP	Coordination Polymer
CP-450	Cytochrome P-450
CZJ	Chemistry Department of Zhejiang University
DCDBP	5,15-bis(2,6-dibromophenyl)-10,20-bis(3,5-dicarboxyphenyl)porphyrin
DPNI	*N,N*ʹ-di(4-pyridyl)-1,4,5,8-naphthalenetetracarboxydiimide
F_10_DPyP	5,15-bis(pentafluorophenyl)-10,20-di(pyridyl)porphyrin
F_5_CPP	5,10,15-tris(*p*-carboxyphenyl)-20-(pentafluorophenyl)porphyrin
F_10_CPP	5,15- bis(*p*-carboxyphenyl)-10,20-bis(pentafluorophenyl)porphyrin
H_8_OCPP	5,10,15,20-tetrakis(3,5-dicarboxylphenyl)porphyrin
MMPF	Metal-Metalloporphyrin Frameworks
MOFs	Metal-Organic Frameworks
PIZA	Porphyrinic Illinois Zeolite Analogue
Por-MOFs	Porphyrin-based MOFs
RPMs	Robust Porphyrinic Materials
SBUs	Secondary Building Units
TNPP	5,10,15,20-tetrakis[*p*-(nicotinoyloxy)phenyl]porphyrin
T*p*CPP	5,10,15,20-tetrakis(*p*-carboxyphenyl)porphyrin
TON	Turnover Number
ZJU	Zheijang University
